# Supplementary material for: A set of multi-entry identification keys to African frugivorous flies (Diptera, Tephritidae)
Source: Zookeys. 2014 Jul 24;(428):97–108. doi: 10.3897/zookeys.428.7366 (PMC4143993; doi:10.3897/zookeys.428.7366)
Supplement: Supplementary material 4 — Key to Capparimyia [file zookeys-428-097-s004.zip › SF4_ZooKeys_key to Capparimyia/lucid_player/help/help/whgdata/whlstf6.htm]

Search Words List


**<<**

tick **1** **2**   
**ticking**   
time **1** **2**   
**tips**   
**tool**   
toolbar **1** **2** **3** **4** **5** **6** **7**   
tools **1** **2** **3**   
top **1** **2** **3**   
topic **1** **2** **3** **4** **5**   
**topics**   
**transferred**   
**translated**   
**treat**   
**treated**   
**treats**   
tree **1** **2** **3** **4** **5** **6**   
trees **1** **2** **3** **4** **5** **6**   
**true**   
**truly**   
**try**   
**trying**   
**turbolinux**   
**turned**   
two **1** **2** **3** **4** **5** **6** **7**   
type **1** **2** **3** **4**   
**types**   
**typical**   
  
  
**ulcers**   
un **1** **2**   
**uncertain**   
uncertains **1** **2**   
uncheck **1** **2**   
unchecked **1** **2** **3**   
unchoose **1** **2**   
**understand**   
**unique**   
unix **1** **2** **3**   
unknown **1** **2**   
**unnecessary**   
**unsure**   
**unusually**   
up **1** **2** **3** **4** **5**   
update **1** **2**   
**updates**   
**upper**   
used **1** **2** **3** **4** **5** **6** **7** **8**   
useful **1** **2** **3** **4** **5**   
**useless**   
user **1** **2**   
**username**   
users **1** **2** **3** **4** **5** **6**   
**uses**   
using **1** **2** **3** **4** **5** **6** **7** **8** **9** **10** **11** **12** **13** **14**   
usually **1** **2**   
**utilities**   
**utility**   
  
  
v1 **1** **2**   
**v3**   
**valid**   
value **1** **2** **3** **4**   
values **1** **2**   
**various**   
**vary**   
version **1** **2** **3**   
**versions**   
very **1** **2** **3**   
view **1** **2** **3** **4** **5** **6** **7** **8** **9** **10** **11**   
virtual **1** **2** **3** **4**   
visit **1** **2** **3** **4**   
**visited**   
  
  
**waist**   
**waisted**   
**wasps**   
**waste**   
way **1** **2** **3** **4** **5**   
**ways**   
web **1** **2** **3** **4** **5** **6**   
website **1** **2** **3**   
**welcome**   
**well**   
what **1** **2** **3** **4** **5**   
**whenever**   
**wherever**   
whether **1** **2** **3**   
while **1** **2**   
white **1** **2**   
**whole**   
why **1** **2**   
wide **1** **2** **3**   
**width**   
**widths**   
**wildflowers**   
will **1** **2** **3** **4** **5** **6** **7** **8** **9** **10** **11** **12** **13** **14** **15** **16** **17** **18** **19** **20** **21**   
window **1** **2** **3** **4**   
windows **1** **2** **3** **4** **5** **6** **7**   
**windows1**   
**wing**   
wish **1** **2**   
within **1** **2** **3** **4** **5** **6**   
**word**   
work **1** **2** **3**   
works **1** **2**   
**workspace**   
world **1** **2** **3**   
worst **1** **2**   
**worth**   
would **1** **2** **3** **4** **5**   
**write**   
written **1** **2** **3**   
www **1** **2** **3** **4** **5** **6** **7**   
  
  
x **1** **2**   
**xml**   
xp **1** **2**   
  
  
**y**   
yellow **1** **2**   
**yet**   
your **1** **2** **3** **4** **5** **6** **7** **8** **9** **10** **11** **12** **13**
